# Adaptive Optical Closed-Loop Control Based on the Single-Dimensional Perturbation Descent Algorithm

**DOI:** 10.3390/s23094371

**Published:** 2023-04-28

**Authors:** Bo Chen, Yilin Zhou, Zhaoyi Li, Jingjing Jia, Yirui Zhang

**Affiliations:** Laser Tangshan Key Laboratory of Advanced Testing and Control Technology, School of Electrical Engineering, North China University of Science and Technology, No. 21, Bohai Road, Tangshan 063210, China; zhouyilin@stu.ncst.edu.cn (Y.Z.); leezy@stu.ncst.edu.cn (Z.L.); m13303108502@163.com (J.J.); zyr894416121@163.com (Y.Z.)

**Keywords:** adaptive optics, wavefront sensorless, SPGD

## Abstract

Modal-free optimization algorithms do not require specific mathematical models, and they, along with their other benefits, have great application potential in adaptive optics. In this study, two different algorithms, the single-dimensional perturbation descent algorithm (SDPD) and the second-order stochastic parallel gradient descent algorithm (2SPGD), are proposed for wavefront sensorless adaptive optics, and a theoretical analysis of the algorithms’ convergence rates is presented. The results demonstrate that the single-dimensional perturbation descent algorithm outperforms the stochastic parallel gradient descent (SPGD) and 2SPGD algorithms in terms of convergence speed. Then, a 32-unit deformable mirror is constructed as the wavefront corrector, and the SPGD, single-dimensional perturbation descent, and 2SPSA algorithms are used in an adaptive optics numerical simulation model of the wavefront controller. Similarly, a 39-unit deformable mirror is constructed as the wavefront controller, and the SPGD and single-dimensional perturbation descent algorithms are used in an adaptive optics experimental verification device of the wavefront controller. The outcomes demonstrate that the convergence speed of the algorithm developed in this paper is more than twice as fast as that of the SPGD and 2SPGD algorithms, and the convergence accuracy of the algorithm is 4% better than that of the SPGD algorithm.

## 1. Introduction

Wavefront sensorless sensing technology, as compared to adaptive optics systems with wavefront sensing, not only reduces the complexity and cost of adaptive optics systems, but it also has significant advantages in situations with weak light or a large turbulence amplitude with distant targets. As a result, they have attracted widespread attention for laser technology and laser communication applications [1,2,3,4,5,6,7] and are one of the most investigated topics in adaptive optics research [8,9,10,11,12].

Optimal control algorithms for wavefront sensorless adaptive optical systems have been divided into two categories: modal-based and modal-free. The mathematical modal-based optimization algorithms include the modal method [13,14,15] and the geometric principle method [16,17,18,19]. In theory, the mathematical modal-based method requires the construction of a mathematical model of the system according to basic functions with the objective of faster convergence. However, the method typically requires the calculation and measurement of the parameters associated with the system model beforehand, which hinders its implementation as compared to the modal-free optimization algorithm. Mountain climbing, simulated annealing, augmented learning, particle swarm, genetic algorithm, and stochastic parallel gradient descent (SPGD) are among the most common modal-free algorithms [20,21,22,23,24,25]. Modal-free optimization algorithms are easier to implement because they do not rely on specific mathematical models. The simultaneous perturbation of the stochastic approximation (SPSA) algorithm has the benefits of simple implementation and robustness and is a common control algorithm for wavefront sensorless adaptive optics; it has also been used to enhance stochastic parallel gradient descent. However, the stochastic parallel gradient descent algorithm can converge at an ideal extreme value with a specific probability with accuracy loss, and it exhibits a certain degree of randomness. Such algorithms require multiple iterations to converge, and each iteration requires the image data to be acquired twice. This is very time-consuming, as the number of algorithm iterations determines whether it can be applied to adaptive optics systems operating in real-time.

In this study, single-dimensional perturbation descent and second-order stochastic parallel gradient descent algorithms (2SPGD) are proposed for a wavefront sensorless system in an effort to increase convergence speed, and their convergence characteristics are analyzed by comparing them to the stochastic parallel gradient descent algorithm, the finite-difference stochastic approximation algorithm, and the second-order simultaneous perturbation of the stochastic approximation algorithm (2SPSA) for theoretical analysis. An adaptive optics numerical simulation of a 32-unit deformed mirror and an experimental optics verification system of a 39-unit deformed mirror are constructed in order to evaluate the practicability, convergence speed, and convergence accuracy of the single-dimensional perturbation descent algorithm.

## 2. Principles of Algorithm and Convergence Speed Analysis

### 2.1. Algorithm Principle

The SPGD algorithm is described as follows: let $u^{\left(k\right)}=\left(u_{1},u_{2},\cdots ,u_{n}\right)$ denote the current control voltage of a deformable mirror, *k* denote the current number of iterations, *n* denote the number of deformable mirror drivers, and $J=\;\int \int \;I^{2}\left(x,y\right)dxdy$ denote the performance index. When updating the correction voltage, first generate a random perturbation voltage vector $\Delta u=(\Delta u_{1},\Delta u_{2},\cdots \Delta u_{n})$ that is independent and follows the Bernoulli distribution, and then apply the positive perturbation voltage $u^{+}=u^{\left(k\right)}+\Delta u$ to obtain the positive performance index $J^{+}$. Then, apply the negative perturbation voltage $u^{-}=u^{\left(k\right)}-\Delta u$ to obtain the negative performance index $J^{-}$, and then calculate the gradient $\Delta J=J^{+}-J^{-}$ of the performance index and update the voltage $u^{\left(k+1\right)}=u^{\left(k\right)}+a\Delta J\Delta u$. The variable *a* is the system gain, which is positive when optimized to its maximum value and is negative otherwise. Follow the preceding steps until the performance index *J* is optimal.

The SPGD algorithm was obtained by improving the algorithm of SPSA, and the 2SPSA algorithm was proposed by Spall et al. [26] recently. This paper was inspired by the 2SPSA algorithm and proposes the 2SPGD algorithm. The 2SPGD algorithm is defined as follows:

(1) $$u^{\left(k+1\right)}=u^{\left(k\right)}+a\frac{G^{\left(k\right)}}{{\bar{H}}^{\left(k\right)}},$$

(2) $$G^{\left(k\right)}=\frac{J^{+}-J^{-}}{2\Delta u},$$

(3) $${\bar{H}}^{\left(k\right)}=\sqrt{H^{\left(k\right)}H^{\left(k\right)}+bI},$$

When the function is non-convex, the second-order algorithm is non-convergent [27]; the function of Equation (3) makes the Hessian matrix [28] positive definite, where *I* is the unit matrix. When the second-order gradient is negative, the correction for the *b* smaller coefficient is 0.00006; this prevents division by zero in Equation (1).

(4) $$H^{\left(k\right)}=0.5\left[\frac{G^{\left(k^{+}\right)}-G^{\left(k^{-}\right)}}{2}\Delta u^{-T}+{\left(\frac{G^{\left(k^{+}\right)}-G^{\left(k^{-}\right)}}{2}\Delta u^{-T}\right)}^{T}\right],$$

In Equation (4), $H^{\left(k\right)}$ represents the approximate second-order gradient, $G^{\left(k\right)}$ represents the first-order gradient, $G^{\left(k^{+}\right)}$ represents the positive perturbation derived from $G^{\left(k\right)}$, and $G^{\left(k^{-}\right)}$ represents the negative perturbation derived from $G^{\left(k\right)}$.

The following describes the finite-difference stochastic approximation (FDSA or serial gradient descent algorithm): Generate a perturbation signal $\Delta u=(\Delta u_{1},\Delta u_{2},\cdots \Delta u_{n})$ with the same magnitude and sign across all vectors, apply the perturbation to each driver in turn, and calculate the performance index value to obtain the vector $\Delta J=(\Delta J_{1},\Delta J_{2},\cdots \Delta J_{n})$; thus, this yields the gradient vector ΔJ·Δu for all drivers, and the optimization equation is $u^{k}=u^{k-1}+a\Delta J\cdot \Delta u$.

**Figure 1:**
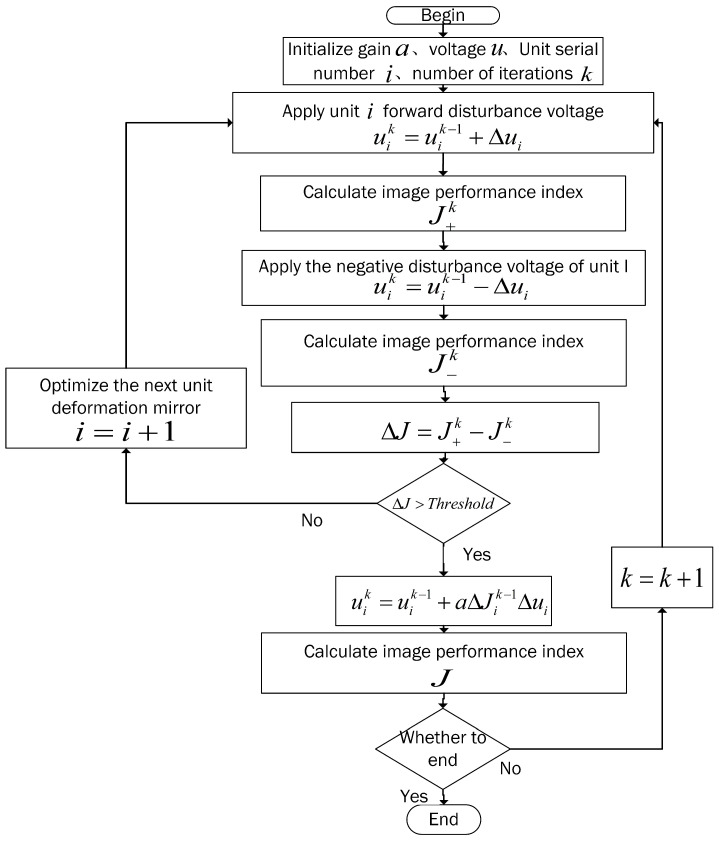
Flowchart of SDPD algorithm.

The coordinate descent method differs from the FDSA algorithm by transforming a high-dimensional optimization problem into a single-dimensional optimization problem and drastically reducing the problem’s complexity. By applying the coordinate descent method, the algorithm for the single-dimensional perturbation descent algorithm(SDPD) is enhanced. The SDPD algorithm’s flowchart is shown in Figure 1. Each step of the SDPD algorithm involves calculating and optimizing the gradient information of a single driving unit of the deformable mirror. When the unit has been optimized to its optimal state, the next unit of the deformable mirror is iterated, and the system is optimized to its optimal state. For instance, step *i* only carries out algorithm optimization for unit *i* of the deformable mirror, applies a positive perturbation voltage $u_{i}^{k}=u_{i}^{k-1}+\Delta u_{i}$ to unit *i* of the deformable mirror, and then calculates the positive performance index $J_{+}^{k}$. Next, we apply a negative perturbation voltage $u_{i}^{k}=u_{i}^{k-1}-\Delta u_{i}$ to unit *i* of the deformable mirror, calculate to obtain the negative performance index $J_{-}^{k}$, and, finally, acquire the gradient information $\Delta J=J_{+}^{k}-J_{-}^{k}$ of unit *i*. When the gradient value ΔJ of the unit is less than the threshold value, it indicates that optimization of the deformable mirror of the unit is complete, and optimization of the next unit is carried out. However, when the gradient value ΔJ of the unit is greater than the threshold value, it is necessary to continue optimization of the deformable mirror of the unit and calculate the updated voltage value of the *i*th unit of the deformable mirror according to Equation $u_{i}^{k}=u_{i}^{k-1}+a\Delta J_{i}^{k-1}\Delta u_{i}$ until all units of the deformable mirror are optimized. Then, the system can complete the wavefront correction. The first step perturbs only the first unit control signal, calculates its gradient, and updates the control signal, i.e., “$u_{1}^{k}=u_{1}^{k-1}+a\Delta J_{1}\Delta u_{1}$”, while the *n*th step perturbs only the *n*th unit control signal, calculates its gradient, and updates the control signal $u_{n}^{k}=u_{n}^{k-1}+a\Delta J_{n}\Delta u_{n}$ until the performance index *J* is optimal.

### 2.2. Analysis of the Convergence Speed of the Algorithm

According to the analysis of Cauwenberghs [29], the SPGD algorithm converges $\sqrt{n}$ times faster, ideally, than the serial gradient descent algorithm (finite difference stochastic approximation algorithm) and $\sqrt{n}$ times slower than the pure parallel gradient descent algorithm, with *n* being the number of system units. The pure parallel gradient descent algorithm can calculate derivatives in the $2^{n-1}$ direction simultaneously as gradients, but it is not applicable to adaptive optical systems. The stochastic parallel gradient descent algorithm computes the gradient in one of the $2^{n-1}$ directions chosen at random. Although the finite difference stochastic approximation algorithm has the same short path as the pure parallel gradient descent algorithm, each iteration needs to measure the derivatives in *n* dimensions in turn, so the iteration speed is *n* times slower than that of the pure parallel gradient descent algorithm.

With the following path analysis, the SDPD algorithm converges faster than the serial method and the SPGD algorithm.

**Figure 2:**
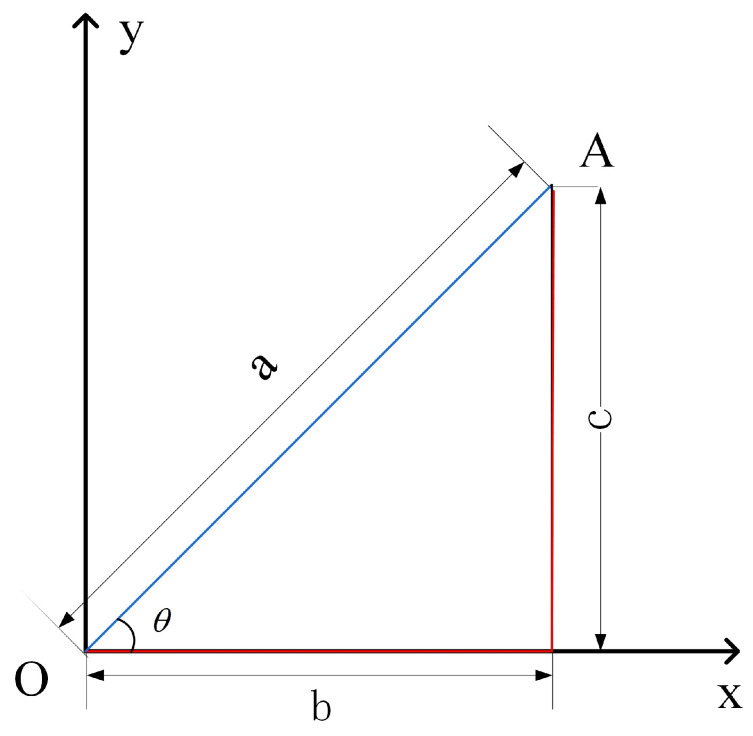
Paths for different algorithms. Point *O* is the starting point of the algorithm, and *A* is the system optimum point; *a* is the distance from point *O* to point *A* for the SPGD, 2SPGD, and FDSA algorithms, and b+c is the distance from point *O* to point *A* for the SDPD algorithm.

In Figure 2, Assuming point *A* is the optimal point of the system and *c* is the pure parallel gradient descent and sequential gradient descent algorithm route, the values of *a*, *b*, and *c* represent the distance traveled by the algorithm; route *L* of the SDPD algorithm is determined, as follows:

(5) L=a+b,

(6) L=c(sin(θ)+cos(θ)),

(7) $$L=\sqrt{2}c\sin\left(\theta +\frac{\pi }{4}\right),$$

(8) $$c\leq L\leq \sqrt{2}c,$$

Generalization to *n* dimensions:

(9) $$L=a_{1}+a_{2}+\cdots a_{n},$$

When $a_{1}=a_{2}=\cdots =a_{n}$, *L* reaches its maximum value:

(10) $$c\leq L\leq \sqrt{n}c,$$

The maximum value of *L* is $\sqrt{n}c$, indicating that the uni-dimensional perturbation descent algorithm is $\sqrt{n}$ times slower than the parallel gradient descent algorithm in the slowest case, while the SPGD is $\sqrt{n}$ times slower than the parallel gradient descent algorithm in an ideal state. Considering that the path of the actual SPGD algorithm contains more randomness and is not necessarily optimal, whereas the path of the SDPD algorithm is fixed and free of randomness, the convergence speed of the SDPD algorithm should be faster than that of the actual SPGD algorithm.

The hill climbing method has the slowest convergence speed and does not utilize the gradient information. FDSA requires *n* iterations and has a slower convergence speed when calculating performance metrics. 2SPGD is a second-order convergence but requires four iterations to calculate the performance metrics, whereas the SDPD and SPGD algorithms only require two iterations. The convergence rates of the SDPD, SPGD, and 2SPGD algorithms are measured experimentally below.

## 3. Experiments and Results Analysis

### 3.1. Numerical Simulation

**Figure 3:**
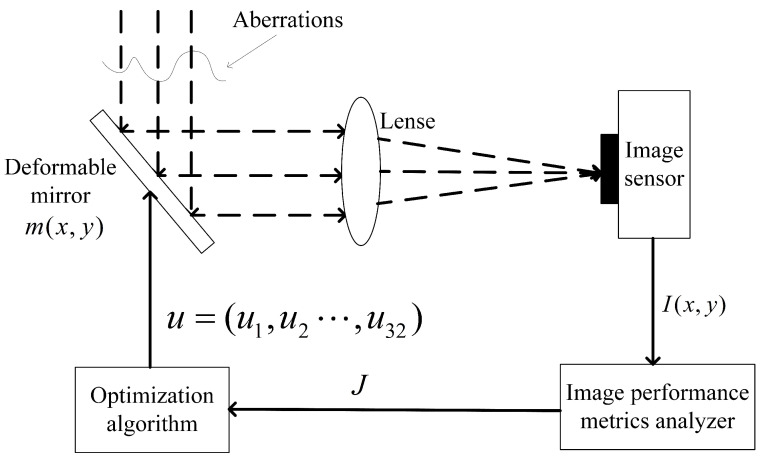
Schematic diagram of the wavefront sensorless adaptive optics numerical simulation. *J* was obtained by analyzing the image sensor data for performance metrics, and then an optimization algorithm (SPGD) was used to generate voltage data to control the deformable mirror.

The simulation model for wavefront sensorless sensing adaptive optics depicted in Figure 3 was established. The performance index analysis module calculated *J* and its gradient ΔJ, and the optimization algorithm calculated the deformable mirror control signal based on *J* and its gradient ΔJ. The system index was optimized through multiple iterations. As the wavefront corrector, a 32-unit deformable mirror was utilized, with the image sharpness function serving as the optimization objective function. The standard Strehl ratio (SR) was computed, and the number of iterations after which the SR reached 0.8 was used as an index to determine the algorithm’s iteration speed. The maximum Strehl ratio value was 1, which indicated no distortion.

The numerical simulation of the deformable mirror was as follows: the phase compensation caused by the deformable mirror is denoted by m(x,y).

(11) $$m\left(x,y\right)=\sum_{i=1}^{32}u_{i}S_{i}\left(x,y\right),$$

(12) $$S_{i}\left(x,y\right)=\exp\left\{\ln w{\left(\sqrt{{\left(x-x_{i}\right)}^{2}+{\left(y-y_{i}\right)}^{2}}/d\right)}^{a}\right\},$$

where (x,y) is the coordinate on the wavefront plane, $\left(x_{i},y_{i}\right)$ is the coordinate on the *i*th unit actuator of the deformable mirror, *w* is the coupling value between actuators and had a value of 0.6, *a* is the Gaussian index and had a size of 2, and *d* is the distance between actuators and had a size of 0.12.

The numerical simulation of the image sensor was performed as follows: the acquired light intensity signal I(x,y) was simulated by a Fourier algorithm.

The image performance index *J* is shown in the following equation:

(13) $$J=\;\int \int \;I^{2}\left(x,y\right)dxdy.$$

The Strehl ratio (SR) is shown in the following equation:

(14) $$SR=\frac{I_{\max}\left(x,y\right)}{I_{\max}^{dl}\left(x,y\right)}.$$

In Equation (14), I(x,y) is the actual distribution of light intensity, $I_{\max}\left(x,y\right)$ is the actual far-field intensity peak, and $I_{\max}^{dl}\left(x,y\right)$ is the ideal distribution of light intensity.

In both the uni-dimensional perturbation descent algorithm and the parallel stochastic gradient descent algorithm, the magnitude of the perturbation voltage and the gradient gain had an effect on the convergence speed; therefore, this paper compares the gain adjustment to the best convergence speed of the respective algorithms under the same perturbation voltage.

### 3.2. Analysis of Simulation Result

Due to the high randomness of SPGD and 2SPGD, an average of 1000 iterations was examined, whereas the single-dimensional perturbation descent algorithm was deterministic and did not require multiple averaging.

**Figure 4:**
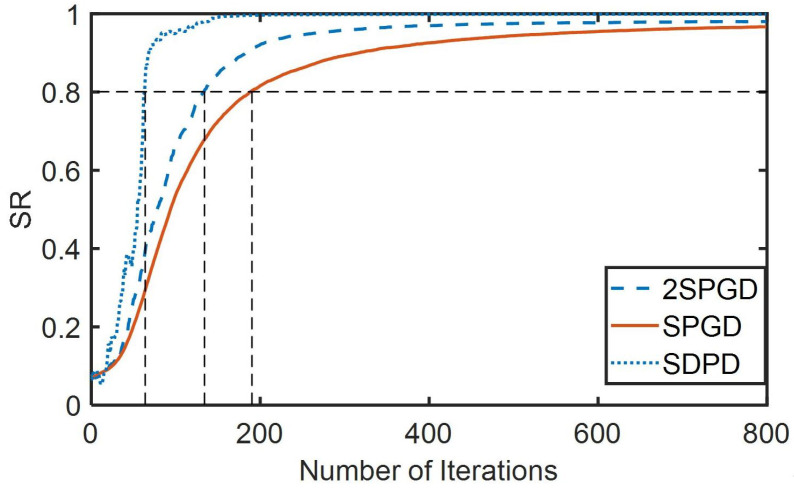
Comparison of the Strehl ratio versus iteration number for the SPGD, 2SPGD, and the SDPD algorithms. The Strehl ratio has been normalized to the maximum.

Figure 4 depicts the simulation results for a 32-unit adaptive optics system. The number of SDPD iterations was greater than the number of 2SPSA iterations, and the number of 2SPGD iterations was greater than the number of SPGD iterations. However, SDPD and SPGD required two measurements per iteration to obtain image data and calculate the performance index, whereas 2SPGD required four measurements per iteration. Therefore, 2SPSA had the worst convergence speed. Table 1 presents the quantitative analysis of the convergence velocity. The SDPD algorithm required 65 iterations to converge to an SR value of 0.8, whereas the SPGD algorithm required 182 iterations, representing a twofold decrease in convergence speed. In contrast to the correction accuracy, the stochastic parallel gradient descent algorithm could only converge to an SR value of 0.958, which was a 4% improvement in accuracy.

In summary, the 2SPGD algorithm had the worst correction effect, while the SDPD algorithm was two times faster than the stochastic parallel gradient descent algorithm, and its convergence accuracy was 4% better than that of the stochastic parallel gradient descent algorithm. In addition, the SDPD algorithm did not require the generation of a random sequence, making it easier to implement than the SPGD algorithm.

### 3.3. Experiments and Results Analysis

**Figure 5:**
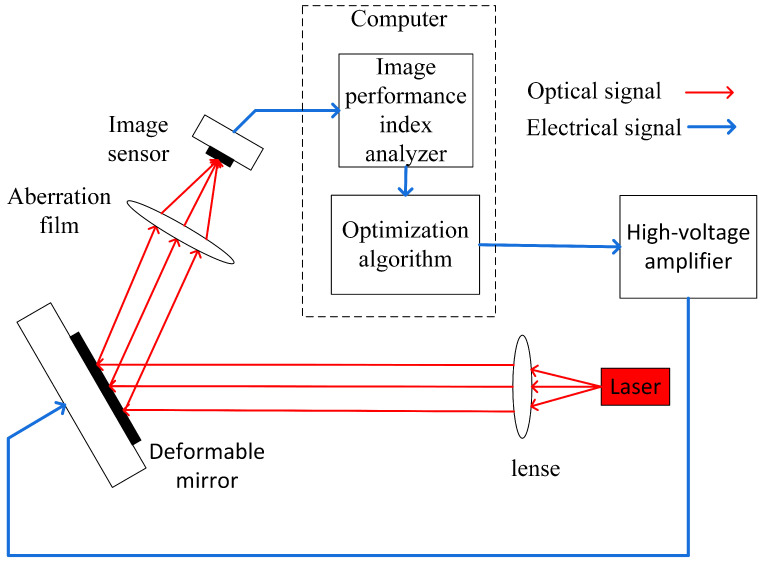
Schematic for wavefront sensorless adaptive optical systems used in the experiments: optimization algorithm were used to correct the wavefront aberrations caused by aberration films. The focal length corresponding to the lens was 18 in. (45.7 cm).

As depicted in Figure 5, a 39-unit adaptive optics experimental setup comprised a laser, a 39-unit micro-electromechanical deformable mirror, a focusing lens, a image sensor, and an computer. The image sensor captured the far-field image, the computer calculated the performance index, and the SDPD and SPGD algorithms controlled the deformable mirror through multiple iterations to complete the wavefront correction. The average results of 100 correction iterations were used to validate the practicability and efficacy of this algorithm.

**Figure 6:**
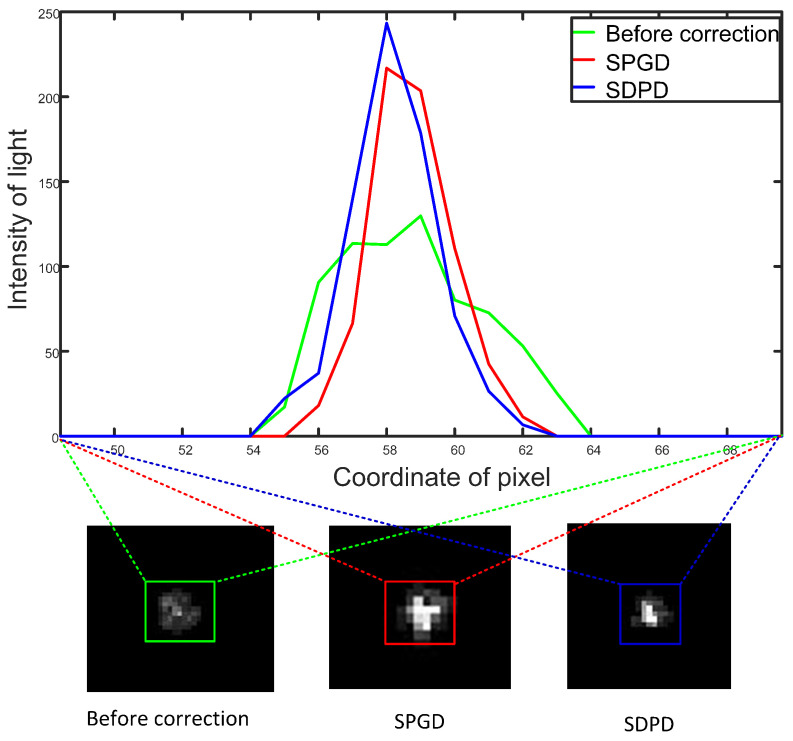
Comparison of the correction effect of SPGD and SDPD algorithms. (**Bottom**) Camera images of the focused spots before and after correction with the SPGD and the SDPD algorithms. (**Top**) spot cross-section data from before and after correction.

**Figure 7:**
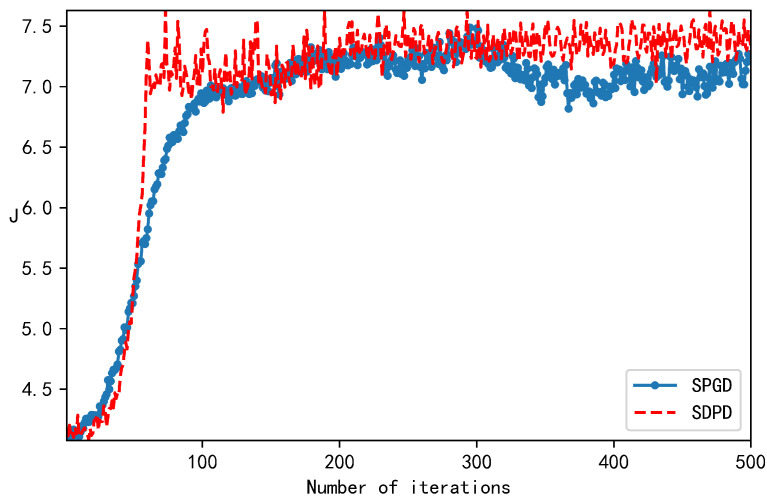
Comparison of *J*-values and number of iterations for SPGD and SDPD algorithms.

Figure 6 (bottom) shows the far-field location prior to and following correction, while Figure 6 (top) shows the far-field spot’s cross-section prior to and following correction. After correction by the SDPD algorithm, it was observed that the far-field spot energy was more concentrated. Figure 7 compares the iteration speed and performance index. The SPGD algorithm converged in approximately 70 iterations, and the SPGD algorithm converged in approximately 160 iterations.

## 4. Conclusions

A SDPD algorithm and 2SPGD were proposed for an adaptive optics system without wavefront detection. The theoretical analysis demonstrated that the convergence rate of the algorithm for SDPD is superior to that of the SPGD algorithm. In the numerical simulation, the SPGD, 2SPGD, and SDPD algorithms were established as the numerical simulation platforms for the controller. The convergence times of SPGD, 2SPGD, and SDPD were 3.185 ms, 4.02 ms, and 0.73 ms, respectively, and the numerical simulation showed that the 2SPGD algorithm was not applicable to the field of adaptive optics, while the SDPD algorithm in the field of adaptive optics had higher real-time performance. The experimental platform further verified the practicability of the SDPD algorithm. The 2SPGD algorithm had fewer iterations, but it had to calculate the performance index four times, so its convergence rate was slower than that of SPGD. 

## Figures and Tables

**Table 1:** Types of algorithms and their convergence effects.

Types of Algorithms	Number of Iterations	Convergence Times	Final Convergence Value of SR
SDPD	65	0.73 ms	0.999
2SPGD	134	4.02 ms	0.958
SPGD	182	3.185 ms	0.957

## Data Availability

https://github.com/Zhou150613/SDPD-vs-SPGD-and-2SPGD.git.

## Abbreviations

The following abbreviations are used in this manuscript:

SDPD	Single-dimensional perturbation descent
SPGD	Stochastic parallel gradient descent
2SPGD	Second-order stochastic parallel gradient descent
SPSA	Simultaneous perturbation of the stochastic approximation
FDSA	Finite difference stochastic approximation
